# The Prosody of Two-Syllable Words in French-Speaking Monolingual and Bilingual Children: A Focus on Initial Accent and Final Accent

**DOI:** 10.1177/00238309211030312

**Published:** 2021-08-04

**Authors:** Margaret Kehoe

**Affiliations:** University of Geneva, Switzerland

**Keywords:** Prosodic development, bilingualism, vocabulary development

## Abstract

This study examined the acoustic characteristics of disyllabic words produced by French-speaking monolingual and bilingual children, aged 2;6 to 6;10, and by adults. Specifically, it investigated the influence of age, bilingualism, and vocabulary on final-to-initial syllable duration ratios and on the presence of initial and final accent. Children and adults took part in a word-naming task in which they produced a controlled set of disyllabic words. Duration and maximum pitch were measured for each syllable of the disyllabic word and these values were inserted into mixed-effects statistical models. Results indicated that children as young as 2;6 obtained final-to-initial syllable duration ratios similar to those of adults. Young children realized accent on the initial syllable more often and accent on the final syllable less often than older children and adults. There was no influence of bilingualism on the duration and pitch characteristics of disyllabic words. Children aged 2;6 with smaller vocabularies produced initial accent more often than children with large vocabularies. Our findings suggest that early word productions are constrained by developmental tendencies favouring falling pitch across an utterance.

## 1 Introduction

French is described as a language with phrase-final accent. Primary stress falls on the final syllable of the last lexical item in a phonological phrase (Dell, 1984). In addition to primary stress, an optional pitch accent may occur on the initial syllable of lexical words (Astésano et al., 2007; Goad & Buckley, 2006; Jun & Fougeron, 1995, 2000, 2002; Post, 2000). Over the years, researchers have gathered much information on “initial accent” in adult speech such that a description of French prosody would not be complete without reference to it (Astésano et al., 2007; Jun & Fougeron, 2000; Post, 2000); however, few studies have investigated initial accent in child speech. We do not know its acoustic properties, nor how optional it is, nor whether its use increases with age.

The aim of this study is to examine word prosody in French-speaking children’s speech with a focus on initial and final accent. We study children aged 2;6 to 6;10 years, allowing us to examine whether the presence of accent varies according to age. The data are collected in Geneva, Switzerland, a city where many bilingual children reside; thus, another aspect of the study is to examine whether bilinguals differ from monolinguals in their realization of accent. We hypothesize that bilingual children who produce initial accent as part of the stress realization of their home language (which is trochaic) will use initial accent in French more often than monolingual children. We also examine whether children’s use of intonation is related to their lexical development (Prieto et al., 2012). In the remainder of the introduction, we discuss initial and final accent in adult speech and prosodic development in French child speech, and complete the section with the research predictions.

### 1.1 Initial and final accent

Descriptions of French prosody refer to two accents: initial and final accent. The final full vowel of lexical words in French receives the primary pitch accent, whereby the domain of accent is the phrase rather than the word. In addition, there is an optional initial accent which falls on one of the first two syllables of a lexical word. Both types of accents are characterized by a fundamental frequency (F0) rise; however, final accent is also associated with duration (Delattre, 1966): the final syllable being approximately 1.8 times longer than the non-final. Final accent has a demarcative function, being associated with the final syllable of a phonological phrase, whereas the function of initial accent is less well understood (Astésano et al., 2007). One of the proposed functions is rhythmic. It breaks up stretches of speech without accents. Another possibility is that it has a demarcative function, signaling the beginning of prosodic structure, namely the prosodic word.

Numerous authors have examined the distribution and conditioning environment of initial accent. Jun and Fougeron (2000) observed that initial accent was realized on the first syllable when a word consisted of two to three syllables, and on the second syllable when a word had more than three syllables. Other factors which condition initial accent include the phonetic nature of the initial segment, the position of the word in the phrase, the length of the phrase, and the morphological nature of the word (D’Imperio et al., 2012; Pasdeloup, 1990; Welby, 2006). Pasdeloup (1990) found it to be present almost all the time on very long words (i.e., 6–7 syllables) and about 30% of the time on short words (3–5 syllables). It varied greatly according to speaker.

Initial and final accent have been integrated into different models of intonation (Hirst & Di Cristo, 1984; Jun and Fougeron, 2000; Post, 2000; Welby, 2006). We consider here the models of Jun and Fougeron (2000) and Post (2000) which both adopt the premises of Autosegmental Metrical (AM) phonology in which an intonation tune is composed of a sequence of underlying high (H) and low (L) tones. Jun and Fougeron (1995, 2000) propose two main intonational units: an accentual (AP) and an intonation phrase (IP). The AP is the lowest tonal unit and is associated with the following tonal pattern /LHiLH*/. The H* associates with the last full syllable of a lexical word, and the Hi associates with the first or second syllable of the AP’s initial lexical word. Thus, H* and Hi are equivalent to what we refer to as final and initial accent. As for the L tones in the AP, the first L tone is realized on the syllable preceding the Hi and the second L tone is realized on the syllable preceding the H* tone. In the case of short words (words of 2–3 syllables), the first L is not always realized and the second L tends to occur in the same syllable as the H*-toned syllable. The IP is the highest unit in the hierarchy and consists of a final boundary tone (L% or H%) which is realized on the last syllable of the IP. When an accentual phrase is final in an intonational contour whose boundary tone is L%, the tonal pattern LHiL may manifest, in which case the accentual tone H* is pre-empted by the IP’s final L%.

In Post’s (2000) account of French intonation, the final accented syllable receives the high starred tone, which can be preceded by a leading H-tone resulting in a bitonal pitch accent H+H*. In this model, H+ and H* are equivalent to what we refer to as initial and final accent. The intonation phrase boundary (beginning and end) can be specified as high (%HH%), low (%LL%) or not be specified for tone (0). An optional low tone (L) may be inserted between the two high tones at the phonological surface level.

An important difference between Jun and Fougeron’s (2000) and Post’s (2000) models is that initial and final accents are distinguished in the first but not in the second account. In Jun and Fougeron’s approach, only the final accent is a pitch accent whereas in Post’s (2000) account, both are considered pitch accents. Another point of difference is the accentual phrase which exists only in Jun and Fougeron’s (1995, 2000) model. The lowest ranked level is the phonological phrase in Post’s (2000) model which is rhythmically or metrically defined and determines where the pitch accents are located. Other authors align themselves with Post’s (2000) account in assuming that initial accent has the same status as final accent and that it is metrically determined (Astésano & Bertrand, 2016; Di Cristo, 2000).

In the current study, we measure initial and final accent in disyllabic words elicited in a picture or object naming task. The target word is always in intonation phrase-final position and is embedded in a short phrase such as un cadeau “a present,” c’est un cadeau “it is a present,” or simply cadeau “present.” The short phrase is equivalent to an accentual phrase in Jun and Fougeron’s, (2000) terminology or a phonological phrase within Post’s (2000) model. It consists of one or more content words and is optionally preceded by one or more function words. It is demarcated by final stress. It cannot be excluded that this context is also one of “neutral” focus since underlyingly there is the notion of an alternative (C’est un cadeau; ce n’est rien d’autre “It is a present; it is not something else”). According to Jun and Fougeron (2000), the intonation contour of a focused utterance is different from that of a neutral utterance. The peak associated with focus (Hf) may be associated with the initial or the final accented syllable. The pitch accents after focus are deleted such that the post-focus sequence is described as “deaccented” (Di Cristo, 1998), and can be modeled as a L tone. In contrast, Post (2000) considers that focal and non-focal accents have the same tonal structure but different degrees of prominence. The acoustic difference is gradual rather than categorical in nature. In this study, we follow Post (2000) in investigating initial accent without making a strong distinction of whether it is serving a focal or non-focal function.

**Figure 1.**
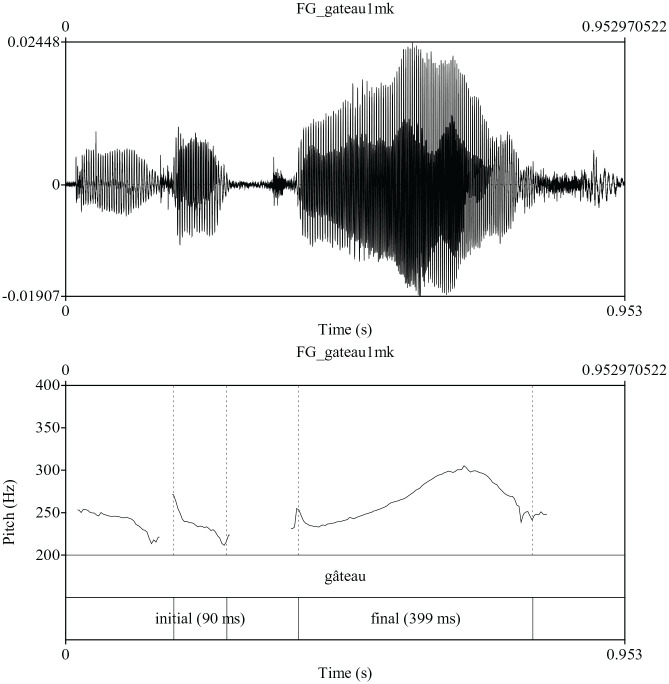
Production of the phrase un gateau /œ̃ gato/ “cake” indicating the presence of final accent only.

**Figure 2.**
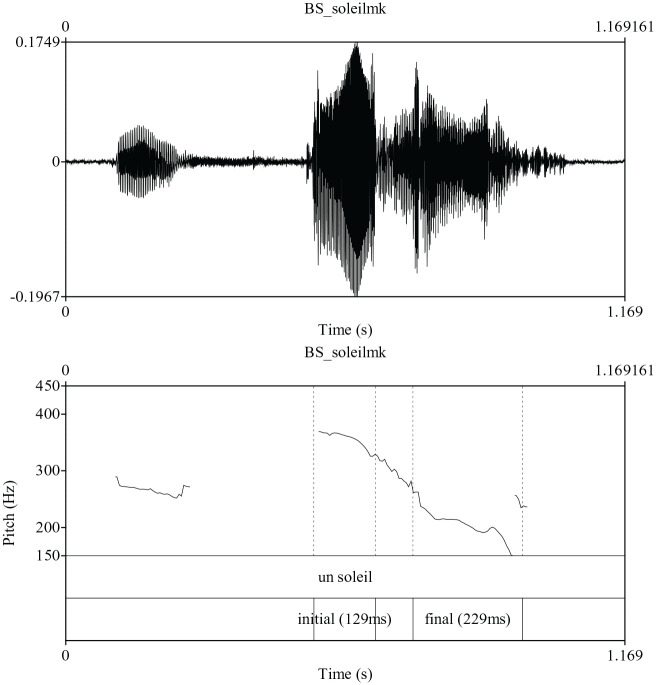
Production of the phrase un soleil /œ̃ solej/ “a sun” indicating the presence of initial accent only.

**Figure 3.**
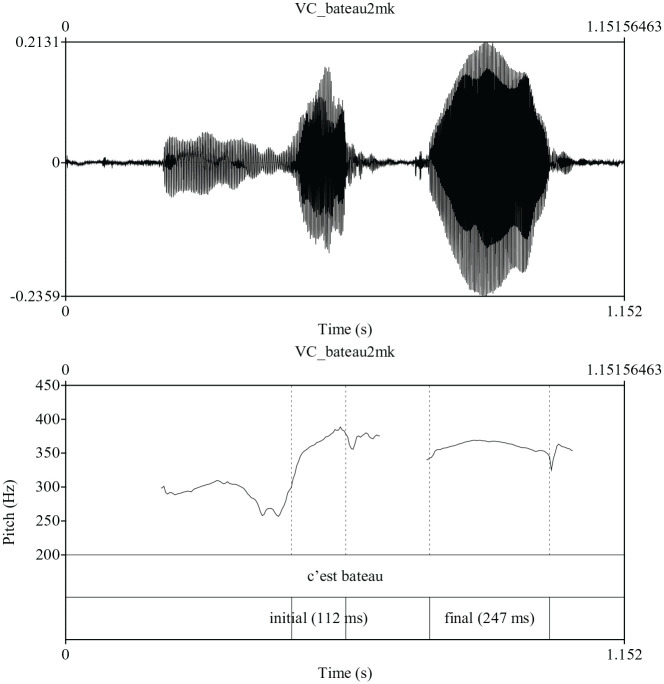
Production of the phrase c’est bateau /se bato/ “that boat” indicating the presence of initial and final accent.

**Figure 4.**
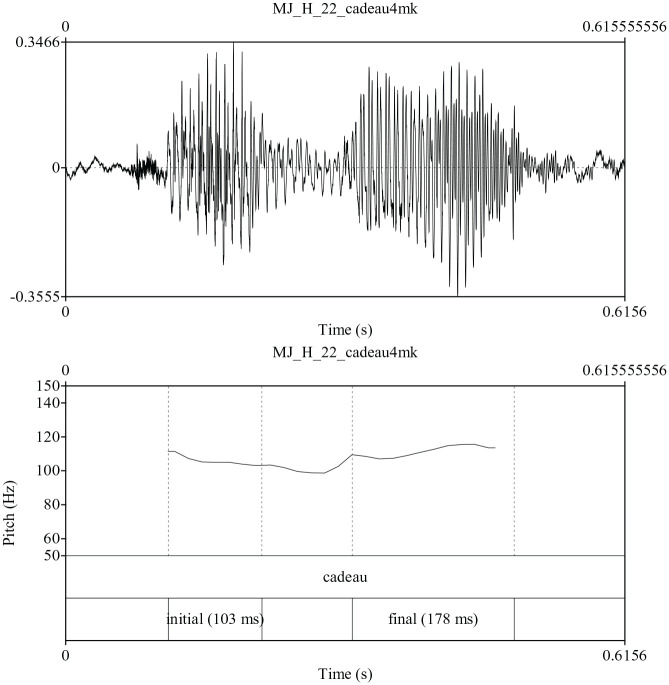
Production of the word cadeau /kado/ “present” realized with little pitch variation. We refer to these productions as non-accented.

Given the above framework, we consider four possible prosodic patterns for the realization of disyllabic target words in the current data. The first is that only final accent is realized. An example is presented in Figure 1. In this case, the final syllable of gateau /gato/ “cake” is characterized by final pitch accent but the optional initial accent is not realized. The second is that only initial accent is realized as indicated by Figure 2, in which the production of soleil /solej/ “sun” is realized with a high tone on the initial but a low tone on the final syllable. Such a pattern may arise because initial accent is, in effect, a focal accent and pitch accents following the focal accent (i.e., final accent) are deleted. Alternatively, final accent may not always be realized in a short accentual phrase which is utterance-final and has a low boundary tone (Jun and Fougeron, 2000). The third pattern is that both initial and final accent are present as suggested by Figure 3. Here the word bateau /bato/ “boat” is realized with two high tones on the first and second syllable. Such patterns were infrequent in the data possibly because the accentual phrases were very short, making it a difficult phonetic task to realize two consecutive pitch accents. Finally, the fourth option is that there is no accentual pattern. Some words were produced in a monotone fashion with little pitch variation. Non-accented productions were observed more often amongst the older children and adults. Figure 4 is an example of cadeau /kado/ produced by an adult in which there is little pitch change across the two syllables. Please note that some authors indicate that final accent may surface even when there is no tonal accent via increased lengthening and that these accents are perceived as metrically strong by listeners (Astésano & Bertrand, 2016). Thus, productions in the “non-accented” category may have final accent realized via duration only. In the next sections, we review studies of French prosodic development.

### 1.2 Prosody in French-speaking monolingual children

Numerous authors have investigated the prosodic development of French-speaking children although most studies have focused on the earliest and not on the latest stages of development (DePaolis et al., 2008; Hallé et al., 1991; Konopczysnki, 1990, 1991; Levitt & Wang, 1991; Vihman et al., 1998; Whalen et al., 1991). Konopczysnki (1990), for example, studied the temporal characteristics of the speech of 12 French babies followed longitudinally from 9 to 24 months. She found that, at around nine months, syllables were equal in duration. After that, the duration of syllables depended upon their position in the utterance. Non-final syllables became shorter and final syllables became longer such that by the age of 14–15 months the duration of final to non-final syllables was around 1.7 similar to the ratio reported for adult speech. Thus, Konopczysnki (1990) claims that the “trailor-timed rhythm” of French is acquired by children in their second year.

Vihman et al.’s (1998) finding support those of Konopczynski (1990, 1991). They studied the acoustic correlates of disyllables produced by 5 French-speaking children at the 25-word point (the first 30-minute session in which infants produce 25 different words). The children were aged from 1;2 to 1;7. Like Konopczynski (1990, 1991), they found that children produced the second syllable longer than the first with a mean duration ratio of 1:1.6. Children also produced higher F0 and amplitude on the second syllable, albeit with considerable variability. Vihman et al. (1998) interpreted their results within a model of the interaction between biological and ambient language factors. They proposed that the natural (biological) tendency for duration is for it to be longer in the second versus the first syllable due to the effects of phrase-final lengthening, which tend to be universal across languages. They assume that F0 and intensity contours decline from the first to the second syllable as a natural consequence of a drop in sub-glottal pressure at the end of a breath group (Lieberman, 1986). Thus, higher pitch and amplitude are more natural in the first than the second syllable. In contrast, French (the ambient language) has phrase-final prominence, which is characterized by more pronounced acoustic correlates (duration, F0, and amplitude) on the second syllable of disyllables (in phrase-final position). Vihman et al.’s (1998) model predicts that when ambient language effects agree with biological tendencies, there should be earlier acquisition of a target prosodic feature; when ambient language effects differ from biological tendencies, there will be later acquisition or children’s productions will be variable. Overall, Vihman et al.’s (1998) findings were consistent with their model. Children acquired the durational features of French easily; however, in the case of pitch and amplitude, where biological and ambient language influences differ, variability was observed. Important to note, however, is that Vihman and colleagues (1998) did not take into consideration the intonation of French in which initial syllables may optionally receive a pitch accent.

More recently, Ménard and colleagues have investigated the acoustic and articulatory correlates of contrastive focus in French-speaking children and adults (Ménard et al., 2006; Ménard et al., 2020; Rapin & Ménard, 2019). Contrastive focus is a type of narrow focus in which the speaker gives emphasis to a specific constituent as opposed to emphasizing another constituent in a paradigmatic comparison (e.g., the word John receives contrastive focus in the sentence “No, I saw John” in response to the question “Did you see Mary?”). Ménard et al. (2020) report that children as young as four years are able to use acoustic correlates, F0, amplitude, and duration, as well as adults when going from a neutral to a focused condition. In contrast, they have not acquired the articulatory correlates of contrastive focus, making less use of lip and finely tuned tongue gestures. Indeed, they find that children have to learn to hypo-articulate in non-emphasized conditions. Although we do not study contrastive focus in the current study, we note that the word-naming task may have generated a type of “neutral” focus, which needs to be taken into consideration.

### 1.3 Prosody in French-speaking bilingual children

Turning to bilinguals, several authors have compared the rhythmic patterns of monolingual and bilingual children using acoustic measurement procedures (Bunta & Ingram, 2007; Kehoe et al., 2011; Mok, 2013; Schmidt & Post, 2015). They have found that bilinguals may differ from monolinguals in the acquisition of rhythm suggestive of the systematic influence of one of the bilingual’s languages upon the other, a phenomenon referred to as crosslinguistic interaction (Paradis & Genesee, 1996). Bilinguals may display patterns of delay in which they distinguish the rhythmic patterns of their two languages at a later time than monolinguals (Mok, 2013; Schmidt & Post, 2015) or they may display patterns of acoustic compromise in which they produce greater vocalic variability in one language and lesser in another resulting in a merging of their two rhythmic patterns (Kehoe et al., 2011). Fewer authors have studied the acoustic correlates of stress (measuring F0 as well as duration) in word productions but some studies exist, namely, those by Rose and Champdoizeau (2007) and Dodane and Bijeljac-Babic (2017) who tested bilingual French-English children.

Rose and Champdoizeau (2007) found that their bilingual participant, aged 2;0 to 3;0 years, already realized the language-specific acoustic characteristics of stress in her two languages. The stressed (initial) syllable versus the unstressed (final) syllable in English was characterized by increased F0 and intensity with minimal duration differences. The stressed (final) syllable versus unstressed (initial) syllable of French was characterized by increased duration with marginal F0 and intensity differences. According to the authors, duration plays the greatest role in signaling stress in French. Pitch is largely irrelevant being implicated in intonation and sentential focus but not in prominence. Thus, Rose and Champdoizeau’s (2007) bilingual child realized stress in her two languages in a similar way to monolinguals.

In contrast, Dodane and Bijeljac-Babic (2017) took both duration and F0 into consideration in their study of word prosody in French-English bilinguals, aged 3;3 to 6;0. They found evidence of cross-linguistic interaction in both acoustic domains. The bilinguals produced overly long final syllables in French, longer even than those of the French monolinguals, possibly to mark the contrast between their English and French productions. They produced high pitch accents on the initial syllable of their French disyllabic productions, more so than they did in their English productions, which the authors interpreted as transfer of the English trochaic F0 pattern to French. Dodane and Bijeljac-Babic (2017) did not entertain the possibility that the high initial tones in the French of the bilinguals may reflect the intonation of French in which a high tone is optionally placed on the initial syllable. Still, they found higher mean F0 values in the initial compared to the final syllable of bilinguals (1.33 semitone difference) relative to monolinguals (.22 semitone difference) suggesting that bilingualism was playing a role in the different prosodic patterns between the two groups.

### 1.4 Prosody and language development

Several authors have observed that growth in intonation may be tied to lexical or grammatical development (Prieto et al., 2012; Snow, 2006). Snow (2006) considers the defining event for intonation development to be the appearance of two-word combinations, which is a grammatical landmark. Others put more emphasis on the relation between intonation and lexical development. Chen and Fikkert (2007) found evidence that intonation development was correlated with vocabulary size in the two-word productions of Dutch-speaking children. Similarly, Prieto et al. (2012) observed that the jump in the number of intonation contours in their Spanish- and Catalan-speaking children was not related to grammatical milestones but to a minimum number of words; all children had passed the 25-word point. Frota et al. (2016) also found intonation to be associated with a jump in lexical development in two Portuguese-speaking children. All of these studies have tested children at the early stages of language development. We are interested in whether there is any association between intonation and vocabulary at later stages.

### 1.5 Summary and research predictions

In sum, several studies have investigated the acoustic correlates of stress in French-speaking children’s disyllabic productions. The findings of Konopczynski (1990, 1991) and Vihman et al. (1998) indicate that the durational correlates of final accent are largely acquired by two years. Few studies, however, have investigated the F0 correlates of initial and final accent in young French-speaking children. Findings on bilingual children acquiring French and English provide a mixed picture of whether bilingualism influences the realization of duration and pitch: some studies find evidence of cross-linguistic interaction (Dodane & Bijeljac-Babic, 2017); others do not (Rose & Champdoizeau, 2007). This study examines the acoustic correlates of French-speaking children’s disyllabic word productions, focusing on the influence of age, bilingualism and vocabulary level on duration and F0 measures. We employ a word-naming task since previous studies have used a similar methodology (Dodane & Bijeljac-Babic, 2017).

#### 1.5.1 Influence of age

To examine the influence of age on the acoustic realization of disyllables, we test children at 3 separate age ranges: 2;6, 3 to 4 (2;11 to 4;11), and 5 to 6 years (5;0 to 6;10) as well as adults. Based on literature findings, we predict that, by 2;6, children will produce final syllables with longer duration than initial syllables and that durational differences will become larger with age. Given the lack of research, we do not make firm predictions on the F0 realization of initial and final accent with age; however, we entertain two possible hypotheses. One possibility is that pitch accent on final syllables is present as of a young age, as suggested by the findings of Vihman et al. (1998), but pitch accent on initial syllables, due to its optional nature, is acquired gradually over time. Another possibility is that initial accent may be present as of a young age because it conforms to the natural biological tendency for pitch to decline across an utterance. In contrast, final accent, being more marked and requiring greater prosodic control, develops over time.

#### 1.5.2 Influence of bilingual status

To examine the influence of bilingualism on the acoustic realization of disyllabic words, we compare monolinguals to two groups of bilinguals: those speaking Romance languages, such as Spanish and Italian, and those speaking Germanic languages, such as English or German. We employ the Speech Learning Model (SLM) of Flege (1995) to characterize patterns of cross-linguistic interaction. Flege (1995) distinguishes two types of processes in bilingual phonetic acquisition: perceptual assimilation and dissimilation. The acquisition of a similar (but not identical) second language (L2) sound may result in equivalence classification which prevents a new L2 category from being formed and as a result the categories of the first language (L1) and L2 are merged together (assimilation). The acquisition of a similar L2 sound may lead to an opposite phenomenon in which the two categories move away from each other to avoid crowding the phonetic space (dissimilation).

Dodane and Bijeljac-Babic (2017) indicate final to non-final ratios of 1.73 for French monolinguals, aged 3;3 to 6;0. A ratio of 1.6 was reported by Vihman et al. (1998) for younger children. In contrast, the final-to-non-final ratio for monolinguals speaking Germanic or Romance languages should be 1.0 or less than 1.0 since their languages are characterized by trochaic stress in which the initial stressed syllable is longer than the final unstressed syllable or at least the same length due to phrase-final lengthening. Delattre (1966) shows that the ratio of stress to unstress is greater in a Germanic than a Romance language. Consequently, we consider the possibility of graded effects in which the final-to-non-final ratio will be smaller in bilinguals speaking Germanic as compared to Romance languages. Thus, we predict that bilinguals speaking Germanic languages will have reduced final-to-non-final ratios compared to bilinguals speaking Romance languages who will in turn have reduced final-to-non-final duration ratios compared to monolinguals. These findings would then be consistent with acoustic compromise in which the ratio is situated in between the monolingual French values and those of the L1 as has been reported in voice onset time (VOT) (Flege, 1991; Flege & Port, 1981) and rhythm (Kehoe et al., 2011) with bilingual children and adults. However, it is also possible that cross-linguistic interaction may lead to a deflecting pattern in which differences become greater to mark the contrast between the two languages, a pattern observed by Dodane and Bijeljac-Babic (2017).

Pitch accent on the initial syllable is an optional feature of French prosody whereas it is an integral part of stress in trochaic languages such as English, German, Spanish, and Italian. If bilingual children are influenced by the prosodic patterns of their L1, we predict that initial pitch accent will be employed more frequently by bilinguals than monolinguals. In a similar vein, we predict that pitch accent on the final syllable should be present more frequently in monolinguals than bilinguals since it is a correlate of final prominence. We do not predict any differences between bilingual children speaking Germanic versus Romance languages with respect to pitch since they all speak trochaic languages. A summary of the research predictions for word prosody is given in Table 1.

**Table 1.** A summary of predictions on cross-linguistic interaction in word prosody. Predictions are based on a comparison of bilingual with monolingual results.

Bilinguals’ L1	Final/Non-final duration ratio	Presence of initial accent	Presence of final accent
Romance	Reduced (weaker)	Greater presence	Lesser presence
Germanic	Reduced (stronger)	Greater presence	Lesser presence

L1: First language.

#### 1.5.3 Influence of vocabulary level

Studies have found that an increase in intonation contours may be associated with vocabulary growth in young Dutch-, Catalan-, Spanish-, and Portuguese-speaking children (Chen & Fikkert, 2007; Frota et al., 2016; Prieto et al., 2012). We examine whether there is any association between the presence of initial and final accent and vocabulary level in older French-speaking children. Once again, due to the lack of research, we do not make firm predictions but entertain several hypotheses. One possibility is that there is no relation between accent and vocabulary acquisition since accent in French is not lexically distinctive. Nevertheless, some authors propose that initial accent may be a marker of the prosodic or lexical word (Astésano et al., 2007; Pasdeloup, 1990; Vaissière, 1991), in which case a second possibility is that initial accent is positively correlated with vocabulary acquisition. A third possibility is that initial accent conforms to the natural biological tendency for pitch to fall across an utterance. In that case, the presence of initial accent may be observed in children with lower vocabulary levels and, thus, be negatively associated with vocabulary development. For the sake of completeness, we examine the influence of vocabulary on the realization of both initial and final accent, including pitch and duration measures.

## 2 Method

### 2.1 Participants

The data come from two studies: Kehoe and Havy’s (2019), in which 40 children, aged 2;6, were tested at the speech laboratory at the University of Geneva; and Kehoe & Girardier’s (2020), in which 101 children, aged 2;11–6;10, were tested at kindergartens or public schools in Geneva. In order to have bilingual groups which were homogenous in terms of L1s (home language other than French) and age, we selected a subsample from these studies. From the Kehoe and Havy (2019) study, we selected 20 children (referred to as group 2;6): 11 children were monolinguals and 9 were bilinguals speaking a Romance language. There were insufficient numbers of bilinguals speaking Germanic languages to form a second group. All children were aged 2;6 (+/- two weeks). From the Kehoe and Girardier (2020) study (referred to as group 3 to 6), we selected 45 children and formed 2 (sub) age-groups: 3 to 4 and 5 to 6 years. In the 3 to 4 group, 8 children were monolinguals, 7 were bilinguals speaking a Germanic, and 7 were bilinguals speaking a Romance language. In the 5 to 6 group, 8 children were monolinguals, 7 were bilinguals speaking a Germanic, and 8 were bilinguals speaking a Romance language. The average age of children was 3;9 in the 3 to 4 group and 5;10 in the 5 to 6 group. A one-way Analysis of Variance (ANOVA) indicated that there were no significant age differences between the three groups at either 3 to 4, *F* (2, 19) = .01, *p* = .99, or 5 to 6 years, *F* (2, 20) = .21, *p* = .81.

In addition, we tested 10 adults (aged 18 to 27 years). All adults were university students. They were monolingual speakers of French who had grown up in French-speaking Switzerland. Some of them also spoke English and German as second-language learners, but they all indicated that they were not proficient speakers of these languages. In sum, the study includes three subgroups of monolingual and bilingual children: aged 2;6, 3 to 4, and 5 to 6 as well as a group of monolingual adults. Although it would have been preferable to include bilingual adults, the focus of the study was on prosodic development in children.

In the 2;6 group, percent exposure to French and to the other language was determined by having the parents complete the Language Exposure Questionnaire (Bosch & Sebastián-Gallés, 1997). Monolinguals were designated as children who received 90 to 100% exposure to French whereas bilinguals were those who received 30 to 80% exposure (mean = 50%). In the older group, bilingual status was based on a questionnaire (loosely based on the PABIQ; Tuller, 2015), in which parents indicated whether their child spoke another language at least 30% of the time in addition to French. Parents were required to judge the language usage of French and the other language on a scale from 1 to 5 (1: only speaks other language; 2: speaks other language more than French; 3: speaks other language the same amount as French; 4: speaks French more than the other language; 5: only speaks French). Because of the small number of children who were dominant in the home language, we formed two dominance groupings: those who were dominant in French (scale 4) and those who were not (scale 2–3). There were more children dominant than not dominant in French particularly in the younger group (3 to 4: Dom = 9; Not dom = 4; 5 to 6: Dom = 8, Not dom = 7). There was missing data on one child. All children had acquired French before the age of three years and, thus, could be considered simultaneous bilinguals.

As for the measurement of vocabulary, parents of children, aged 2;6, completed the L’Inventaire Français du Développement Communicatif (IFDC) (Kern & Gayraud, 2010) (the European French adaptation of the MacArthur-Bates Communicative Development Inventory, MCDI; Fenson et al., 1993) and the MCDI of the child’s other language if the children were bilingual. Thus, we obtained two separate scores: French and Total vocabulary. The number of words in the IFDC is 688, whereas the number of words in the MCDI of the other language was variable. In the case of the older group, children were administered a French vocabulary test (EVALO2-6; Coquet et al., 2009). We used a restricted set of the EVALO2-6 (minus the body parts) yielding a total score of 54. We did not administer a vocabulary test in the child’s home language. For both vocabulary measures, we calculated raw rather than standardized scores given the difficulties of applying standardized scores to bilingual children.

Information on the children’s percent language exposure/dominance, languages spoken, and vocabulary level is presented in Tables A.1 and A.2 in Appendix A. As Table A.2 indicates, the languages spoken by the Germanic group included English, German, Swiss German, Norwegian, and Swedish and the languages spoken by the Romance group were Catalan, Italian, and Spanish. In some cases, children were trilinguals speaking two different languages at home. We ensured that the main language spoken at home (by the mother) was a Germanic or Romance language.

### 2.2 Stimuli

The stimuli for the children included (roughly) 16 disyllables selected from a pool of words produced by children during an object or picture naming task as well as a memory game (see Kehoe & Girardier, 2020, and Kehoe & Havy, 2019, for further details). The majority of stimulus words can be found in the IFDC and/or in the Developpement du langage de production en français (DLPF) version 3 (31–36 mois/months) (Bassano et al., 2005). A slightly different set of words was employed with the younger and older children, since the children were tested in two separate studies with slightly different aims. The adults took part in a separate study again and produced disyllabic words as part of a memory game (Kehoe & Kannathasan, 2021). The set of words for the 2;6 group, the 3 to 6 group, and the adults is shown in Table 2.

**Table 2.** Disyllabic words produced by children aged 2;6 and 3 to 6 and by adults.

Words produced by children aged 2;6	Words produced by children aged 3 to 6	Words produced by adults
**cadeau** ^a^	**bateau**	**bateau**
**cerise**	**cadeau**	**cadeau**
chemise	**cerise**	**cochon**
**cheval**	**cheval**	**dauphin**
**dauphin**	**cochon**	**gâteau**
**fenêtre**	**dauphin**	**guitare**
**fromage**	**fenêtre**	
garçon	**fromage**	
**girafe**	**gâteau**	
**grenouille**	**girafe**	
**lunette**	**grenouille**	
poulet	guitare	
requin	**lunette**	
**salade**	**rideau**	
**soleil**	**salade**	
tambour	**soleil**	

a. Words in bold were produced by at least two of the age groups.

Segmental content of the target words may influence prosodic measures (particularly duration). Therefore, several precautions were taken to ensure that segmental content did not confound prosodic measures:

1. Every attempt was made to select the same set of words for each child in a given group.

2. Target words were coded for the vowel quality of the first and second syllable and for the syllable structure of the second syllable; these variables were included as control variables in the statistical models.

3. Item (i.e., the target word) was included as a random factor in the statistical model.

### 2.3 Procedure

Both groups of children took part in a production task of approximately 20 to 30 minutes in which they were encouraged to name pictures and objects of the stimulus words. The children were asked “Qu’est-ce que c’est?” (What is that?) or “Comment ça s’appelle?” (What is that called?) The 3 to 6 group also played a memory game in which the child had to find a pair of the same picture by remembering where the picture was situated within an array of pictures. The child was required to name the pair of pictures each time they had a turn allowing us to obtain multiple repetitions of a given picture. Group 2;6 was tested in the speech laboratory at the University of Geneva and Group 3 to 6 was tested in a quiet room in the children’s kindergarten or school. Children in Group 2;6 interacted with a native French-speaking experimenter and, on occasion, one of their parents, whereas children in Group 3 to 6 interacted with two native French-speaking experimenters. The testers were instructed to elicit spontaneous productions of stimulus words but, when this was not possible, to obtain productions through imitation (see analyses on imitation below). Children in both data sets produced on average 18 disyllabic words (2;6: *SD* = 3.8, range = 12–25; 3 to 6: *SD* = 2.3, range = 13–23). Adults were tested in a quiet room on the university campus. They played the same memory game that the older children played. They were requested to say the words as they normally would when playing a memory game. Adults produced on average 21 disyllabic words (*SD* = 3.0, range = 18–25).

### 2.4 Data analyses

The children’s and adult’s productions were recorded with a portable digital tape-recorder (MARANTZ, TASCAM DR-2d) and unidirectional condenser microphone placed on a table in front of them. Using Phon, a software program designed for the analysis of phonological data (Rose & MacWhinney, 2014), each child’s and adult’s WAV file was segmented, and stimulus words were identified and transcribed. Four French-speaking graduate students, who had experience in phonetic transcription, performed the segmentation. Disyllabic words were extracted for the analysis of word prosody.

Acoustic analyses were conducted in Praat (Boersma and Weenink, 2016). To measure the acoustic correlates of disyllables, we focused on the vocalic nucleus of each syllable. The onset and offset of each vowel were designated as the first and last detectable periodic cycle in the time waveform.^1^ Inspection of the time waveform and spectrogram as well as auditory judgment was used to aid boundary identification. Once the vocalic nucleus of each syllable was defined, we extracted the following measures: duration of initial and final syllables; and maximum pitch of initial and final syllables. We then calculated: (a) the duration ratio = duration of final/duration of initial syllable; and (b) pitch difference in semitones between the maximum pitch of the initial and final syllables. The latter was calculated automatically using the f2st function in R. The presence of pitch accent was defined as a 1.5 semitone difference in (maximum) pitch between the first and second syllable. According to Rietveld and Gussenhoven (1985), differences of 1.5 semitones are perceptually salient. A 1.5 semitone difference corresponded roughly to a 30Hz difference between initial and final syllables, which was equivalent to a 10% change in F0, the average maximum F0 of the first syllable in children being 300 Hz. A positive difference indicated the presence of initial accent and a negative difference, the presence of final accent. See Figures 1 and 2 for examples of final and initial accent.

**Figure 5.**
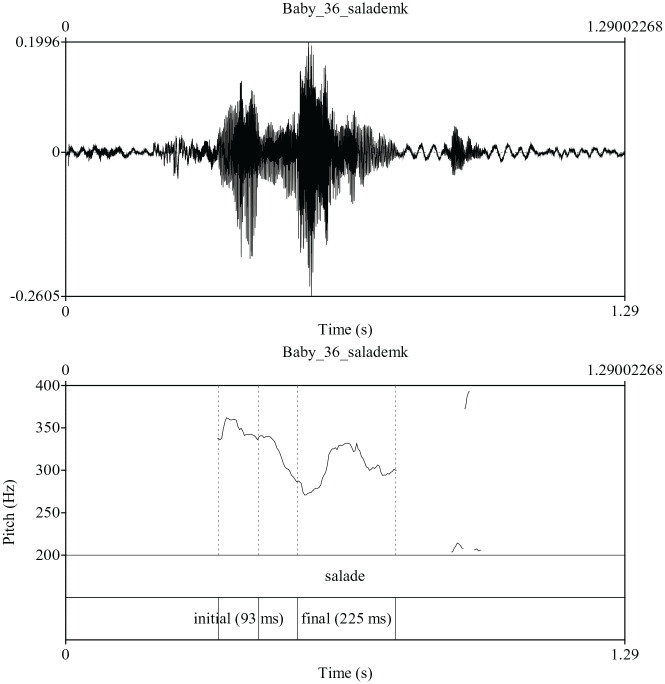
Production of the word salade /salad/ “salad” indicating the presence of initial and final accent. When no function word was present, initial-final accent was determined by presence of pitch excursion in one or both of the syllables.

The presence of productions containing both initial and final accent was determined qualitatively by “eyeballing” pitch displays of each individual word or phrase. We considered a production to have initial and final accent when both syllables of the disyllable were characterized by a high tone in the presence of a low tone on the preceding function word (see Figure 3). In the case of productions in which no function word (hence low tone) was present, the presence of a rise-fall tone on one or both syllables of the disyllable was also indicative of initial and final accent (see Figure 5).

### 2.5 Reliability

A subsample of words (*n* = 156 from 8 different children; approximately 11% of the data) was reanalyzed acoustically applying the same procedure as described above. The analyses were conducted by students who had experience in acoustic analysis. The mean absolute duration difference between the first and second analysis was 7.29 ms (*SD* = 7.99) for syllable 1 and 8.32 ms (*SD* = 10.10) for syllable 2. The mean absolute pitch difference was 1.69 Hz (*SD* = 2.34) for syllable 1 and 2.56 Hz (*SD* = 3.9) for syllable 2. The correlation coefficient between the two sets of duration scores was .96 for syllable 1 and .99 for syllable 2; the correlation coefficient between the two sets of pitch values was .99 for syllable 1 and .99 for syllable 2. In addition, a subsample of words (*n* = 115 from 10 different children and adults) was re-examined perceptually in order to determine whether the word was characterized by an accent on both syllables or not. Inter-rater reliability was 87% (110/115 words perceived by 2 judges as having accent on both syllables vs. accent on the initial or final syllable). Overall, the acoustic and perceptual reliability tests suggest acceptable inter-tester reliability.

### 2.6 Data-coding

One potential confound was the presence of imitated productions which were more frequent in the younger than in the older children’s productions and which were absent from the adult productions. An imitation was defined as a production by a child in which an adult production of the same target word directly preceded it. Productions in which there was an intervening phrase or a temporal delay (greater than 2000 ms) were not counted as imitations. Children may have employed initial (or final) accent because it was used by the adult to elicit the production. To circumvent this confound, all productions were coded as to whether they were imitated or spontaneous and, if they were imitated, the adult production was also coded as to whether it contained initial or final accent or no accent. Imitation was used as a control variable in the statistical models which involved the analysis of pitch.

Another potential confound was that both children and adults produced a given target word multiple times with adults producing greater numbers of repetitions than children. To ensure that there was no effect of repetition on the acoustic characteristics of the word productions, we coded productions in terms of whether they were the first or a repeated production of the target word. The variable “repetition” was included in the statistical model.

Finally, we coded whether the target word was preceded by one or more function words (yes or no) since pronouncing words in isolation or in a short phrase may potentially alter the accentual pattern on the lexical word. The variable “function word” was included in the statistical models which tested pitch accent.

Before statistical analysis, outliers were removed. Outliers were those values which exceeded 2.5 standard deviations of the mean value of the duration ratio and F0 difference as defined for each subject. In the child data, 24 outliers were removed for pitch values (2;6: *n* = 7; 3 to 4: *n* = 10; 5 to 6: *n* = 7) and 22 outliers were removed for duration (2;6: *n* = 8; 3 to 4: *n* = 6; 5 to 6: *n* = 8). No outliers were removed in the adult data.

### 2.7 Statistical analyses

The analyses were performed using R statistical software (R Development Core Team, 2020) and the lme4 package (Bates et al., 2015) for mixed models. Duration measures were analyzed using mixed models (lmer function) whereas pitch measures were analyzed using binomial logistic regression (glmer function). Comparisons were made using likelihood ratio tests (LRT) which yield a chi-squared statistic. To determine differences between groups, we employed Tukey multiple comparisons (emmeans function). Please note that we employed the lmer function in the case of duration because we were interested in the magnitude of the duration ratio whereas we employed the glmer function in the case of pitch because we were interested in whether children realized an accent or not (see Kehoe and Kannathasan, 2021, and Stoehr et al., 2018, for a similar approach when measuring VOT lag or lead).

Fixed effects were age, coded into four groups 2;6, 3 to 4, 5 to 6, and adults, and bilingual status.^2^ In the analysis of duration, bilingual status included three levels: monolingual, bilingual-Romance (bi-Rom), bilingual-Germanic (bi-Ger), whereas in the analysis of pitch, bilingual status included two levels: monolingual and bilinguals. To distinguish the two variables, one having three levels and one, two levels, we employ the term “bilingual type” for the three-level variable. As noted above, we predicted some graded differences between bilinguals speaking Germanic and Romance languages in terms of duration but not in terms of pitch accent. In addition to the predictor variables, there were several control variables: vowel1 and vowel2, which took into consideration the vowel quality of the initial and final vowels of the disyllable; and CVC2, which took into consideration whether the final syllable was open or closed (gâteau vs. salade). Vowel1 was coded into four levels: schwa (e.g., cerise /səʁiz/ “cherry”), high vowel (e.g., lunettes /lynɛt/ “glasses”), mid vowel (e.g., cochon /koʃɔ̃/ “pig”), and low vowel (e.g., cadeau /kado/ “present”). Vowel2 was coded into three levels: high vowel (e.g., chemise /ʃəmiz/ “shirt”), mid vowel (e.g., gâteau /gato/ “cake”), and low vowel (e.g., salade /salad/ “salad”). All things being equal, low vowels are longer than mid vowels which are longer than high vowels due to intrinsic vowel length effects. Schwa is generally shorter than a full vowel. Vowels in open syllables are longer than in closed syllables. The vowel quality and syllable structure variables were included only in models which tested duration. In contrast, the variables, imitation and function word, which coded whether the target word was preceded by an adult imitation or was produced with a function word (e.g., bateau vs. un bateau), were only employed in the analysis of pitch. In both duration and pitch analyses, we included the control variable, repetition (first vs. repeated production) to determine whether repeating a word influenced its acoustic realization.

To test the effect of vocabulary level on pitch and duration measures, we ran separate models on the younger (i.e., 2;6) and older (i.e., 3 to 6) groups. This is because the two groups were tested with different vocabulary measures which were not comparable. To remind the reader, we had a measure of French and Total Vocabulary based on the MCDI for the younger children, which is a measure of the number of words that they produce, whereas we had a vocabulary score in French for the older children, which is the number of words they know on a French vocabulary test. After the age of 2;6 months, it is considered an impossible task to keep a track of the number of words children produce (Feldman et al., 2005), thus necessitating the use of two different vocabulary measures.

## 3 Results

### 3.1 Influence of age and bilingual status/type

#### 3.1.1 Duration

The first model examined the effect of age and bilingual type on the final to initial duration ratio while controlling for vowel quality, syllable structure, and repetition. There were 1364 individual items in the model. Results showed that there was a significant effect of age, *χ*^2^(3) = 14.60, *p* = .002, but not of bilingual type, *χ*^2^(2) = .23, *p* = .89, on duration ratios.^3^ Tukey multiple comparisons (with corrections applied) indicated that children aged 2;6 had significantly lower duration ratios and children aged 3 to 4, had marginally lower ratios than children aged 5 to 6 (2;6: *t* = -3.57, *p* = .003; 3 to 4: *t* = -2.45, *p* = .076). Apart from the findings on age, analyses indicated that the vowel quality of the initial and final vowel influenced duration ratios and the syllable structure of the final syllable marginally influenced them, vowel1: *χ*^2^(3) = 11.75, *p* = .008; vowel2: *χ*^2^(2) = 10.65, *p* = .005; CVC2: *χ*^2^(1) = 3.53, *p* = .06. Whether a production was a repetition or not did not influence duration measures, *χ*^2^(1) = .28, *p* = .60.

Table 3 shows the means and standard deviations (*SD*s) of the duration of the initial and final syllable of the disyllable, and the duration ratio (final/initial) according to the four age groups: 2;6, 3 to 4, 5 to 6, and adult. As can be seen, the duration of syllables becomes shorter over time (with the exception of differences between 2;6 and 3 to 4). The duration ratio ranges from 1.64 to 2.04, displaying a tendency to become larger across age in the child data but then to pull back in the adult data to 1.77.

**Table 3.** Means and standard deviations (*SD*s) of syllable durations and duration ratios for the three age groups of children (2;6, 3 to 4, 5 to 6) and for the adults.

Age	Duration initial		Duration final		Duration ratio final/initial		
	Mean	*SD*	Mean	*SD*	Mean	*SD*	*n*
2;6	139.81	49.56	210.00	89.88	1.64	.84	343
3 to 4	142.46	49.13	237.06	112.34	1.74	.81	404
5 to 6	107.46	39.40	204.96	99.16	2.04	1.02	388
Adult	96.25	25.29	166.47	75.87	1.77	.79	207

Table 4 presents the means and standard deviations of the duration ratios according to bilingual type for the child data. Children aged 2;6 are displayed separately from the older children since there were no Germanic bilinguals amongst the youngest group. Adults are not included because they were essentially monolinguals. As suggested by the statistical analyses, in which no significant effect emerged, duration ratios were similar across monolingual and bilingual children.

**Table 4.** Means and standard deviations (*SD*s) of duration ratios for the children according to bilingual status.

	Age 2;6			Age 3 to 6		
	Duration ratio final/initial			Duration ratio final/initial		
	Mean	*SD*	*n*	Mean	*SD*	*n*
Mon	1.62	.87	182	1.90	.90	280
Bi-Rom	1.66	.79	161	1.85	.92	261
Bi-Ger				1.91	.98	251

Mon: monolinguals; Bi-Rom: bilinguals speaking Romance languages; Bi-Ger: bilinguals speaking Germanic languages.

#### 3.1.2 Pitch

Before conducting statistical analyses, we first examined the number of productions of target words which were imitated. The youngest children’s productions were characterized by a high percentage of imitations (30.77% or 108/351 productions) whereas the older children’s productions contained a smaller percentage (3 to 4: 6.83% or 28/410; 5 to 6: 2.53% or 10/396). Adults had no imitations. The adult production preceding the child production was almost always accented (143/146 cases) and, in the majority of cases, it was characterized by initial accent (72.73% or 104/143 productions). We also examined the percentage of target word productions preceded by one or more function words. The youngest children produced on average 42.45% of words (149/351) accompanied by a function word, whereas the older children produced a greater percentage of target words accompanied by function words (3 to 4: 76.59% or 314/410; 5 to 6: 73.23% or 290/396). The adults did not use function words as their data were collected as part of another project (on VOT measurement; Kehoe & Kannathasan, 2021) in which the use of bare nouns was encouraged.

The second statistical model examined the effect of age and bilingual status on the presence of pitch accent while controlling for whether the production was imitated or spontaneous, whether it was a repetition or not (first or repeated production), and whether it was preceded by a function word (yes or no). We used binomial logistic regression and ran two separate models, one for initial and one for final accent. Results indicated that there was a significant effect of age on both the presence of initial and final accent, initial: *χ*^2^(3) = 24.53, *p* < .001; final: *χ*^2^(3) = 23.19, *p* < .001. Tukey multiple comparisons (with corrections applied) indicated that children aged 2;6 produced initial accent significantly more often than children aged 3 to 4 (*z* = 2.66, *p* = .04), children aged 5 to 6 (*z* = 4.51, *p* < .001) and adults (*z* = 4.01, *p* < .001). Children aged 2;6 produced final accent significantly less often than children aged 5 to 6, (*z* = -3.21, *p* = .007) and adults (*z* = -3.05, *p* = .01). In addition, children, aged 3 to 4 produced final accent significantly less often than children aged 5 to 6 (*z* = -4.00, *p* < .001) and adults (*z* = -3.43, *p* = .003). Bilingual status was not significant, initial: *χ*^2^(1) = .03, *p* = .87; final: *χ*^2^(1) = 1.67, *p* = .20, nor was the effect of imitation, initial: *χ*^2^(1) = 1.01, *p* = .87; final: *χ*^2^(1) = 06, *p* = .80^5^, repetition, initial: *χ*^2^(1) = .0004, *p* = .99; final: *χ*^2^(1) = .16, *p* = .69, or function word, initial: *χ*^2^(1) = .32, *p* = .57; final: *χ*^2^(1) = 1.09, *p* = .30.

We also examined the influence of age and bilingual status on the percentage of productions judged to have accent on both syllables. To remind the reader, we used perceptual judgment (visual and auditory) to determine whether both initial and final accent was present. Our statistical model revealed that neither age or bilingual status contributed to model fit: age: *χ*^2^(3) = 3.39, *p* = .34; bilingual status: *χ*^2^(1) = .11, *p* = .74, nor were the control variables (i.e., imitation, repetition, function word) significant.

Table 5 presents the descriptive findings on pitch. It shows the means and standard deviations of the maximum F0 of initial and final syllables and the mean semitone difference between the two syllables. As would be expected, raw F0 values decline across age due to biological changes in vocal fold mass and length. Mean semitone differences are in favor of higher pitch on the initial syllable at the two younger ages and slightly higher pitch on the final syllable at the two older ages.

**Table 5.** Means and standard deviations (*SD*s) of maximum pitch and pitch differences (in semitones) for the three age groups of children and for the adults.

Age	Max. F0 initial		Max. F0 final		Semitone difference initial-final		
	Mean	*SD*	Mean	*SD*	Mean	*SD*	*n*
2;6	374.66	78.53	333.88	73.14	1.99	2.42	344
3 to 4	318.86	67.03	297.43	62.39	1.20	2.42	400
5 to 6	287.56	43.45	293.09	63.03	–.15	3.69	389
Adult	165.81	62.36	168.93	59.93	–.50	2.90	207

F0: fundamental frequency.

Table 6 shows the mean percentage of productions which were designated as having initial accent, final accent, and initial-final accent across age group and bilingual status. The percentage of productions which were characterized by little pitch variation across both syllables (see Figure 4) was sizeable (18 to 30% across age-group). Thus, we examined the proportion of productions which were not tonally marked but which were nevertheless characterized by long final syllables. As indicated above, some authors consider duration may be sufficient to trigger accent perception (Astésano & Bertrand, 2016). We used a final-to-initial duration ratio of 1.7 to separate productions with long final syllables and productions without. The percentage of these production across age group and bilingual status is shown in Table 6 (column labeled “duration only”). Finally, the “other category” refers to productions which were not tonally marked according to the 1.5 semitone difference or perceptual criteria or which were not characterized by long final syllables.

**Table 6.** The mean percentage (and standard deviations, *SD*s) of productions realized with initial accent, final accent, initial-final accent, “duration only” accent, and “other” according to age and bilingual status.

	*n*	Initial		Final		Initial-Final		Duration only		Other	
		Mean	*SD*	Mean	*SD*	Mean	*SD*	Mean	*SD*	Mean	*SD*
Age 2;6											
Mon	184	68.11	14.99	5.68	5.61	8.80	10.71	6.36	6.35	11.75	9.44
Bi	160	62.09	23.32	9.99	8.72	8.40	7.13	8.39	9.53	11.20	9.61
Age 3 to 4											
Mon	143	37.40	20.54	11.07	8.35	13.49	9.83	20.02	17.86	18.03	14.94
Bi	257	46.46	21.02	4.76	7.60	13.03	21.35	17.93	13.54	17.81	16.19
Age 5 to 6											
Mon	131	32.69	18.65	14.36	23.45	14.70	11.36	24.69	16.88	15.02	13.16
Bi	258	30.33	20.32	33.62	25.25	13.81	12.02	12.37	13.18	9.88	9.11
Adults											
Mon	207	22.48	19.51	29.89	12.51	16.68	9.83	13.17	8.79	17.77	13.56

Mon: monolingual; Bi: bilingual.

The table shows that the percentage of words with initial accent declined across age whereas the percentage of words with final accent increased. The percentage of words with both initial-final accent was low across all age groups but tended to increase with age (from 8% to 17%). The percentage of words marked by duration only was low at the youngest age but reached percentages between 12% and 25% at later ages. The percentage of productions which did not receive any tonal or “duration only” accent ranged from 10% to 18%. The table also reveals some minor differences between monolinguals and bilinguals. For example, bilinguals aged 5 to 6 produced more words with final pitch accent but fewer words with “duration only” accent than monolinguals; however, as indicated, the statistical analyses revealed no effect of bilingualism on the realization of accent. In sum, we observe that, from the youngest age range (2;6), children realize the final syllable with longer duration than the initial syllable. However, only the older children and adults realize the final syllable with a pitch accent. The younger children realize the pitch accent predominantly on the initial syllable. There was no effect of bilingual status/type on duration ratios or on the presence of pitch accent. Apart from initial or final accent, both children and adults realize a small proportion of productions with initial-final accent and with a “duration only” accent.

### 3.2 Influence of vocabulary level

**Figure 6.**
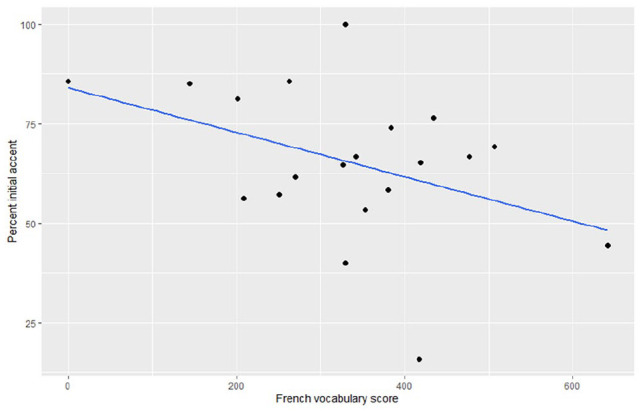
Scatterplot of the relation between percent initial accent and French vocabulary level in children aged 2;6.

To examine the influence of vocabulary on the final-to-initial duration ratios and on the presence of initial and final pitch accent, we ran separate models for children aged 2;6 and children aged 3 to 6 years. In the case of the younger children, we examined the effect of French and Total vocabulary on the duration ratios while controlling for vowel quality, syllable structure, repetition, and bilingual type, and we examined the effect of vocabulary on pitch accent while controlling for bilingual status, imitation, repetition, and function word. Age was not included as all children were of similar age. Results indicated that neither French nor Total vocabulary levels influenced duration ratios, French: *χ*^2^(1) = .03, *p* = .87; Total: *χ*^2^(1) = 1.60, *p* = .21. French vocabulary level did, however, influence the presence of initial, *χ*^2^(1) = 6.94, *p* = .008, but not final accent, *χ*^2^(1) = .66, *p* = .42. Children with low vocabulary levels realized initial accent more frequently than children with high vocabulary levels. Total vocabulary level did not influence the presence of initial, *χ*^2^(1) = 1.06, *p* = .30, nor final accent, *χ*^2^(1) = .002, *p* = .97. Figure 6 displays a scatterplot of the relation between percentage initial accent and French vocabulary for children aged 2;6.

In the case of the older children, we conducted the same analysis examining the effect of French vocabulary on final-to-initial duration ratios and on initial and final accent. The children varied in age and, thus, alongside control variables, age (in months) was included. French vocabulary did not have a significant effect on duration ratios, *χ*^2^(1) = .08, *p* = .78, nor on the presence of initial, *χ*^2^(1) = 1.72, *p* = .19, and final accent, *χ*^2^(1) = .40, *p* = .53. In contrast, age had a significant effect on the realization of initial and final accent, initial: *χ*^2^(1) = 6.97, *p* = .008; final: *χ*^2^(1) = 11.63, *p* < .001, as was found previously in the statistical model which included children and adults. In sum, vocabulary knowledge influenced the presence of initial accent in the youngest but not in the oldest group of children. It did not influence the presence of final accent nor duration ratios in either age group.

### 3.3 Additional analyses with dominance

Our analyses indicated that bilingual status/type did not influence the duration or pitch realization of disyllabic words. We reconducted the analyses examining whether coding the data in terms of language dominance (monolingual, dominant in French, not dominant in French) rather than bilingual status (monolingual, bilingual) would change the results. We focused only on the child dataset. In the 2;6 group, four bilingual children were dominant (60% or more exposure rate to French) and five (50% or less exposure rate to French) were not dominant in French. In the 3 to 6 group, 17 children were dominant and 11 were not dominant in French as determined by a rating scale completed by the parents (see section 2 Method).

Results indicated that dominance did not emerge as significant in models testing the influence of predictor variables on duration, *χ*^2^(2) = .92, *p* = .63, or pitch, initial: *χ*^2^(2) = .15, *p* = .93; final: *χ*^2^(2) = 3.82, *p* = .15, suggesting that even bilingual children who were not dominant in French had acoustic realizations of disyllabic words similar to that of monolingual children. A summary of the duration and pitch results across the different analyses is given in Table 7.

**Table 7.** Summary of the duration and pitch results.

	Duration	Pitch	
Variables	Final/Non-final ratios	Initial accent	Final accent
Age	Children aged 2;6 had smaller duration ratios than children aged 5 to 6 but not adults	Children aged 2;6 had higher presence of initial accent than older children and adults.	Children aged 2;6 and 3 to 4 had lower presence of final accent than older children and adults.
Bilingual status/type (or dominance)	No influence	No influence	No influence
Vocabulary level	No influence in younger or older children	Children aged 2;6 with lower vocabulary levels displayed greater presence of initial accent. No influence in older children.	No influence in younger or older children.

## 4 Discussion

This study investigated the presence of initial and final accent in monolingual and bilingual French-speaking children and in monolingual French-speaking adults. Specifically, we investigated the influence of age, bilingual status/type, and vocabulary knowledge on final-to-initial duration ratios and on the presence of initial and final accent in disyllabic words. Our results indicated strong effects of age on the realization of pitch accent and lesser effects of age on duration measures; there was no influence of bilingual status/type on either duration and pitch measures. Vocabulary level influenced the realization of pitch in the younger children’s productions. In the following paragraphs, we summarize the findings and discuss their relevance to our understanding of pitch accent in French-speaking children.

### 4.1 Influence of age

Our findings confirm what has been reported before: that, from an early age, children have acquired the “trailor-timed” pattern of French rhythm (Konopczysnki, 1990; Vihman et al., 1998). The youngest children produced final syllables longer than initial syllables (ratio = 1.64) and their ratios were not found to be statistically different from those of adults (1.77). They had significantly lower ratios relative to the oldest group of children (i.e., 2.04); however, the pattern of the older children may reflect “overshoot” since their values were greater than those of adults (2.04 vs. 1.77). “Overshoot” patterns have been reported in other temporal domains such as VOT, whereby children display a tendency to produce highly aspirated stops before realizing them with adult-like values (Ryalls & Larouche, 1992; Splendido, 2017). In sum, the duration characteristics of final accent are well established from an early age. These findings are also consistent with the durational characteristics of initial and final accent in French. Astésano (2001) reports that initial and final accent differ in intra-syllabic durational dimensions: the onset is longer than the rhyme for initial accent whereas the rhyme is longer than the onset for final accent. Syllables with initial accent are only slightly longer than unaccented syllables. Our measurement procedure, which focused on vocalic nuclei only, would have captured the long rhymes of final accent.

In contrast to the findings with duration, the presence of pitch accent on the final syllable was not present from an early age. It was observed significantly less often in the younger compared to the older children and adults. Even in the older children and adults, it was present only 28% of the time. The low presence of final accent is consistent with reports which indicate that final stress in French is more strongly cued by duration than pitch. It may also reflect the nature of the task; that is, word naming, which may have been of low interest to the older participants and have elicited pitch accent less well than other types of tasks might have done. In addition, the accentual phrase was in utterance-final position, and, thus, final accent may have been overridden by the low boundary tone typical of a declarative utterance.

Initial accent was present to a high degree in the youngest children’s productions (i.e., 62 to 68% across monolinguals and bilinguals; see Table 6) and to a moderate degree in children aged 3 to 4 (i.e., 37 to 46%). One of our hypotheses was that initial accent, being an optional aspect of French prosody, should be acquired gradually over time. This was not found to be the case. The other hypothesis we entertained was that initial accent may be present from an early age because it conforms to the natural biological tendency for pitch to decline across a breath group (Lieberman, 1986; Vihman et al., 1998). Numerous studies report a predominance of falling over rising contours in children’s early speech patterns (Behrens & Gut, 2005; Chen & Kent, 2009; Lleó et al., 2004; Snow, 2006). Falling tones have been found to outnumber rising tones in early word productions (Behrens & Gut, 2005; Chen & Fikkert, 2007); to be substituted for rising tones more frequently than the reverse (Lleó et al., 2004; Snow, 1998); to be easier to imitate than rising tones (Loeb & Allen, 1993); and to be produced in elicitation tasks more often than rising tones (Patel & Brayton, 2009; Patel & Grigos, 2006). In sum, the preponderance of initial accent in the younger versus older children may reflect biological tendencies which favor falling over rising contours (Vihman et al, 1998).

Initial accent was present to a lesser degree in older children and adults (22 to 33%) when naming words. However, we also examined the presence of pitch accent when adults attempted to elicit words from children. There were 146 examples in the current database and, in the majority of cases, adults used either initial or final accent to elicit a production from the child (143/146). Initial accent was considerably more present than final accent (104/143 vs. 39/143). Thus, we observe that adults do employ initial accent frequently in certain pragmatic contexts. In this case, initial accent probably served as a focal accent to highlight the word to elicit its production. We cannot exclude that the higher presence of initial accent in the younger children may reflect the fact that initial accent served as focal accent. Ménard et al. (2006) also reported the presence of an unwanted focus in a neutral condition when designing an elicitation task for young children. An alternative (but not necessarily incompatible) explanation is that young children are more likely to engage in pointing, whether it is gestural or vocal (prosodic), and initial accent may be an expression of this pointing action (Loevenbruck et al., 2009). Other authors point to the fact that initial accent has an expressive function which may also be consistent with its higher use in younger children (Vaissière, 1991). A more phonological account comes from Shattuck-Hufnagel et al. (1994) who report a tendency by adults to place accent early in a word or phrase to indicate that a new intonational phrase has begun. Such a tendency may be even more pronounced in young children. In sum, several factors may be responsible for the greater presence of initial accent in the younger versus older children which include biological tendencies, novelty of the task, the presence of focal accent, or prosodic pointing.

Word productions judged to have initial-final accent were present to a small degree in the data (i.e., 8 to 17%). They increased roughly with age (see Table 6), although our statistical model revealed no significant age effect. We believe that one of the reasons for their low frequency was the shortness of the words (i.e., disyllables). Authors have observed that the presence of both initial and final accent increases as word length increases (Pasdeloup, 1990).

### 4.2 Influence of bilingual status/type

Our analyses indicated no influence of bilingual status/type on the duration and pitch realization of children’s disyllabic productions. In terms of duration, we hypothesized that children who speak a trochaic language will produce smaller final-to-initial duration ratios than monolingual children, consistent with acoustic compromise between the duration values of their L1 and that of French. We also hypothesized that there may be gradient effects between bilingual children who speak Germanic versus Romance languages due to the purported differences between stressed and unstressed syllables in these languages. Instead, we observed no influence of bilingual type on duration ratios neither in statistical models which included all age groups or which focused on the children separately.

In terms of pitch accent, we hypothesized that bilingual children would produce pitch accent more frequently on the initial and less frequently on the final syllable than monolingual children, because initial accent conforms to the stress pattern of the L1 which is trochaic. We observed no differences between monolingual and bilingual children in the frequency at which they realized initial and final accent. Overall, our findings are consistent with Rose and Champdoizeau (2007) who found no evidence of cross-linguistic interaction in the word prosody of their bilingual English-French child. They are different from other studies which have reported cross-linguistic interaction in word prosody (Dodane & Bijeljac-Babic, 2017) or in rhythm (Bunta & Ingram, 2007; Kehoe et al., 2011; Schmidt & Post, 2015). Kehoe and Kannathasan (2021), employing a database similar to the current one, documented an influence of bilingual status on VOT but only when dominance was taken into consideration. Bilingual children who were not dominant in French had positive VOT values that differed from monolinguals whereas bilingual children who were dominant in French did not differ. We conducted additional analyses which took language dominance into consideration, yet did not find an influence of dominance on word prosody. Overall, these results suggest that bilingual status/type has little influence on the acoustic realization of words when children are exposed to their two languages at a young age.

### 4.3 Influence of vocabulary level

Several studies investigating intonation in young children have observed a relationship between grammatical, vocabulary, and intonation development (Chen & Fikkert, 2007; Frota et al., 2016; Prieto et al., 2012). We aimed to determine whether such a relationship exists in slightly older children, focusing exclusively on vocabulary. Nevertheless, accent in French is not lexically determined, with the exception of initial accent, which some authors claim to be a marker of the lexical word (Astésano et al., 2007). Thus, we posited that vocabulary level may not have an influence on the realization of accent, or, if it does, only on initial accent. Our hypotheses were essentially confirmed. Vocabulary level did not influence duration ratios or the presence of final accent. It influenced the presence of initial accent in the youngest group of children. However, the presence of pitch accent was negatively correlated with vocabulary level. Children with low French vocabulary levels produced more initial accent. It is likely that vocabulary score here provides an indication of developmental level and the higher presence of initial accent in the low vocabulary children (i.e., in the less developmentally advanced children) is consistent with the notion that initial accent reflects a biological tendency for pitch to fall across an utterance. The high vocabulary children (i.e., more developmentally advanced group) appear to have overcome this biological tendency. It is surprising that only French and not Total vocabulary influenced the presence of initial accent; however, some missing data were present in the Total vocabulary measures (see Kehoe & Havy, 2019) and consequently French vocabulary may have been a more reliable indicator of vocabulary level than Total vocabulary. In the older children, age was a stronger determinant of whether children employed initial (and final) accent than vocabulary.

### 4.4 Understanding initial accent

One of the principal aims of the study was to provide information on the presence of initial accent in child speech. Indeed, we are unaware of any previous study which has investigated initial accent in a young French-speaking population (with the exception of studies by Ménard et al. (2006, 2020), which have studied contrastive stress). We observed that despite its optional nature in adult speech, it is employed frequently by young children and its presence diminishes over time. Amongst the adults, it was present 22% of the time which is not very different from the value reported by Pasdeloup (1990) for the presence of initial accent in the production of short words by adults (i.e., 30%). We hypothesize that its increased presence in the younger children is due to the fact that initial accent conforms to the natural biological tendency for pitch to fall across an utterance and possibly also to the nature of the task, which was more novel for the younger compared to the older participants. We observed that final accent was rarely employed by the younger children, suggesting that it is indeed a phonetically more complex structure and is only produced with adult-like frequency toward the age of 5 to 6 years.

In the introduction, we contrasted two alternative models of intonation: Jun and Fougeron (2000) and Post (2000). Although the central aim of the study was not to test these two theoretical approaches, certain findings in the data, such as the high presence of initial accent in the young children’s productions and the association (albeit negative) of initial accent with vocabulary are consistent with a model of intonation in which initial accent has an important role in the demarcation of prosodic structure and a similar status to that of final accent, as in the framework of Post (2000).

Admittedly, the findings of this study are limited to the extent that they measure initial accent in one pragmatic context and it is unclear whether these results generalize to other pragmatic contexts and to spontaneous speech. We have also been unable to tease apart whether initial accent is serving a focal function or is serving a variety of functions, focal, rhythmic, and other. Experimental studies as well as corpora data would be needed to compare the presence of initial accent when clearly expressing a contrastive/corrective function (Ce n’est pas une carotte. C’est une marmotte!), versus in a word-naming task, as in the current study, or in spontaneous speech. It would also be important to elicit multi-phrase productions in which initial and final accent are not always situated in utterance-final position and thus may be pre-empted by the final boundary tone. Finally, we acknowledge that we employed quantitative criteria to determine initial and final accent and qualitative criteria to determine initial-final patterns and a more uniform methodology across all accentual patterns would be preferable in future analyses.

The acquisition of initial accent is of theoretical interest due to its optional nature. How do children acquire structures which are optionally cued in the input? We can compare the acquisition of initial accent to that of schwa in French which is frequently deleted in adult speech (e.g., cerise [səʁiz]/[sʁiz]) and, thus, is optionally present in the input. Findings on the acquisition of schwa in French show that children are more likely to produce the variant with schwa even when they are exposed to input forms in which schwa is absent (Andreassen, 2013). Similarly, in the current study, children produced forms with initial accent more often than it appears to be present in adult speech. Application of Andreassen’s (2013) findings on French schwa to the current data leads us to expect that, after a period in which children’s speech is subject to phonological or developmental constraints which result in a high degree of initial accent production, children’s speech would then be subject to the stylistic and phonetic constraints that are operative in adult speech. Further research of a longitudinal nature is needed to confirm when and how children’s realizations of initial accent approximate the patterns of the adult input.

## 5 Conclusion

This study examined the presence of initial and final accent in disyllabic words produced by children and adults in a word-naming task. We found that the durational features of final accent were present from an early age but not the accentual features. Young children more frequently realized an accent on the initial than final syllable and, with increasing age, the relative proportion of initial and final accent resembled more closely that of adult speech, which was characterized by greater accent realization on the final syllable. We did not find any differences between monolingual and bilingual children in their realization of the duration and pitch parameters underlying initial and final accent. Young children with low vocabulary realized initial accent more frequently than children with high vocabulary levels, consistent with our hypothesis that initial accent reflects a developmental constraint for pitch to decline across an utterance. However, alternative explanations such as its role as a prosodic pointer may underlie its high presence in young children. This study represents a first attempt to document the presence of initial accent in young French-speaking children. Additional research of an experimental and longitudinal nature is needed to track its presence in different pragmatic contexts and over time.

## Supplemental Material

sj-pdf-1-las-10.1177_00238309211030312 – Supplemental material for The Prosody of Two-Syllable Words in French-Speaking Monolingual and Bilingual Children: A Focus on Initial Accent and Final Accent Click here for additional data file. Supplemental material, sj-pdf-1-las-10.1177_00238309211030312 for The Prosody of Two-Syllable Words in French-Speaking Monolingual and Bilingual Children: A Focus on Initial Accent and Final Accent by Margaret Kehoe in Language and Speech

## Notes

1. In the case of words grenouille /gʁənuj/ “frog” and soleil /solej/ “sun,” the vowel plus glide /j/ counted as vocalic nucleus of the final syllable since it was difficult to segment the vowel only.

2. We coded age into discrete levels rather than leaving it as a continuous variable because the data were heterogenous: children in the youngest group were all the same age (within +/- two weeks), and the oldest group (i.e., the adults) had high values for age if coded in months. In the analysis which just focuses on the 3 to 6 group (for testing the effect of vocabulary), age (in months) is used.

3. Since the adults were coded as monolinguals, we also repeated the analysis just including the children. Bilingual status/type did not contribute to model fit in this analysis, either when coded in terms of a three-way distinction (mon, bi-Rom, bi-Ger; *χ*^2^(2) = .21, *p* = .90) or a two-way distinction (mon, bi; *χ*^2^(1) = .013, *p* = .91).
