# Supplementary material for: The Prosody of Two-Syllable Words in French-Speaking Monolingual and Bilingual Children: A Focus on Initial Accent and Final Accent
Source: Lang Speech. 2021 Aug 4;65(2):444–71. doi: 10.1177/00238309211030312 (PMC9014682; doi:10.1177/00238309211030312)
Supplement: sj-pdf-1-las-10.1177_00238309211030312 – Supplemental material for The Prosody of Two-Syllable Words in French-Speaking Monolingual and Bilingual Children: A Focus on Initial Accent and Final Accent [file sj-pdf-1-las-10.1177_00238309211030312.pdf]

Appendix A. 1. Information on the monolingual participants in group 2;6 and 3 to 6, including gender, age, %exposure to French (group 2;6 only), and vocabulary level.

Participant	Gender	Age	% French	Vocab
Child 30	f	2;6	90	342
Child 35	m	2;6	90	353
Child 51	m	2;6	90	641
Child 8	m	2;6	100	329
Child 22	m	2;6	100	270
Child 26	m	2;6	100	384
Child 27	f	2;6	100	251
Child 31	m	2;6	100	419
Child 32	f	2;6	100	434
Child 34	m	2;6	100	201
Child 38	f	2;6	100	507

Participant	Gender	Age		Vocab
Age 3 to 4				
RL	m	2;11		--
MR	m	3;3		46
GH	m	3;4		32
IA	m	3;5		24
VM	m	3;9		34
SL	m	4;3		52
MS	f	4;8		48
DWL	f	4;9		36
Age 5 to 6				
BM	f	5;1		52
LA	m	5;4		48
MI	f	5;4		54
DB	m	5;7		42
GN	f	5;10		50
BS	m	6;1		48
GM	f	6;3		46
VE	m	6;6		54

Note: Vocabulary level for the 2;6 group is based on parent report (maximum score = 688) whereas vocabulary level for the 3 to 6 group is based on results of a French vocabulary test (maximum score = 54).

Appendix A. 2. Information on the bilingual participants in group 2;6 and 3 to 6, including gender, age, %exposure to French/dominance, languages spoken, and languages spoken by respective parents, where L2 was learned and age of French onset (for group 3 to 6), and vocabulary level in French.

Participant	Gender	Age	% French	L1/L1+	L1 spoken by	Fr Vocab	Tot. Vocab
Child 43	m	2;6	30	Spanish	both parents	0	552
Child 44	f	2;6	35	Spanish	mother	144	389
Child 48	f	2;6	35	Spanish	father	380	458
Child 50	m	2;6	40	Spanish	both parents	208	648
Child 36	f	2;6	50	Italian	both parents	377	544
Child 6	f	2;6	60	Spanish	father	417	496
Child 14	m	2;6	60	Spanish	both parents	263	263
Child 33	f	2;6	60	Italian/Spanish	both parents	477	477
Child 17	m	2;6	80	Spanish	father	329	329

Participant	Gender	Age	Dominance	L1/L1+	L1 spoken by	L2 learned at	Age of French onset	Vocab
Age group: 3 to 4								
Germanic Language								
CI	M	2;11	Dom	English	mother	home	0	22
AL	M	3;1	Dom	English	father	home	0	26
WJ	M	3;2	Not dom	German	mother & father	crèche	2;9	12
BC	F	3;4	Dom	Norwegian	mother	home	0	48
OL	F	4;3	Dom	English	father	home	0	48
VC	F	4;6		English	mother	home	0	54
DGY	F	4;11	Dom	German	mother	home	0	40
Romance Language								
CE	M	3;0	Not dom	Italian	mother& father	crèche	2;6	22
DL	M	3;4	Not dom	Italian/Dutch	mother/father	crèche	0;8	18

AL	M	3;5	Dom	Spanish	mother & father	crèche	2;0	2
MA	F	3;6	Dom	Spanish	mother	home	0	26
DC	M	3;9	Dom	Italian	mother	home	0	34
BS	F	4;7	Not dom	Italian	mother/father	crèche	0;5	32
DL	F	4;8	Dom	Catalan	father	home	0	50
Age group: 5 to 6								
Germanic Language								
LC	M	5;1	Not dom	German/Swedish	mother/father	crèche	2;0	26
OPA	F	5;4	Not dom	English	mother & father	crèche	2;4	14
JL	F	5;9	Dom	English/Bosnian	mother/father	home	0	22
BFG	F	6;1	Not dom	Swedish/Farsi	mother/father	crèche	2;0	26
BM	F	6;3	Dom	German	mother	home	0	52
FG	M	6;4	Dom	German/Spanish	mother/father	home	0	40
IS	F	6;7	Not dom	Swiss German	mother & father	crèche	2;0	40
Romance Language								
PK	M	5;1	Dom	Spanish	mother	home	0	36
RN	F	5;4	Dom	Spanish	mother	home	0	48
VM	F	5;7	Not dom	Italian	mother & father	activities	3;0	30
LA	F	5;9	Dom	Spanish	grandparents	home	0	36
AA	F	5;11	Dom	Italian/Spanish	mother/father	crèche	2;6	44
FA	M	6;1	Dom	Italian	mother/father	home	0	48
RA	F	6;1	Not dom	Spanish	mother/father	crèche	2;0	52
HD	M	6;7	Not dom	Spanish	mother/father	crèche	2;0	34

Note: Vocabulary level for the 2;6 group is based on parent report (maximum score for French = 688) whereas vocabulary level for the 3 to 6 group is based on results of a French vocabulary test (maximum score = 54).
